# Speed and Blood Parameters Differ between Arabian and Žemaitukai Horses during Endurance Racing

**DOI:** 10.3390/ani11040995

**Published:** 2021-04-01

**Authors:** Indrė Poškienė, Renata Gruodytė, Jurgita Autukaitė, Vida Juozaitienė, Ramūnas Antanaitis

**Affiliations:** 1 Large Animal Clinic, Veterinary Academy, Lithuanian University of Health Sciences, Tilžės St. 18, LT-47181 Kaunas, Lithuania; renata.gruodyte@lsmuni.lt (R.G.); jurgita.autukaite@lsmuni.lt (J.A.); ramunas.antanaitis@lsmuni.lt (R.A.); 2 Department of Animal Breeding, Veterinary Academy, Lithuanian University of Health Sciences, Tilžės St. 18, LT-47181 Kaunas, Lithuania; vida.juozaitiene@lsmuni.lt

**Keywords:** exercise, horse, endurance, pace, horse breed

## Abstract

**Simple Summary:**

Limited information exists on the physiological changes that occur in the horses competing in endurance races. The objective was to provide the initial data describing changes in laboratory measurements of the horses competing in endurance races under temperate conditions and to compare the data between the Arabian horses, which are one of the most popular horse breeds in the world, and a Lithuanian horse breed—Žemaitukai. The study was carried out on 112 horses. Blood samples were collected before and after an endurance race. The Arabian horses were faster compared to the local breed (Žemaitukai). The study showed significant changes in horse blood gasometrical and biochemical indicators.

**Abstract:**

Fédération Equestre Internationale (FEI) has described equine endurance racing as the second largest discipline in the world, above which is only show jumping. The Žemaitukai is an ancient indigenous Lithuanian horse breed known since the 6th or 7th century. The Arabian horse breed is one of the oldest human-developed horse breeds in the world. Compared with other race horse breeds, the muscle tissue of Arabian horses is characterized by significant differences in structure—a predominance of oxidative fiber type I is observed in Arabians, making them the prevailing breed in endurance racing. The Arabian horses are recognized as the leading breed in endurance competitions. Speed, pace, and total time in the race strategy have been extensively studied in human sports, and in contrast, this strategy appears to have been virtually ignored in equestrian sport, despite the potential for contributing to performance optimization. In relation to speed and total time in the race, there are limited data on postrace physical, biochemical, and blood gas parameters of endurance horses. Thus, this study was carried out to investigate the effects of speed on the blood parameters of the Arabian and Žemaitukai horses during an endurance race. Blood samples were taken before and immediately after the exercise. Biochemical and blood gas indicators were analyzed. The study showed significant increases in mean blood gasometrical indicators, such as partial carbon dioxide pressure (8.09–15.18%, *p* < 0.001); base excess in the extracellular fluid (14.01%, *p* < 0.001 in the Arabian horses and 172.01% in the Žemaitukai breed, *p* = 0.006); decreases of the blood electrolyte ionized calcium (4.38–8.72%, *p* < 0.001) and the hematocrit and hemoglobin values (20.05–20.12%, *p* < 0.001 in the Arabian horses and 6.22–6.23% in the Žemaitukai breed, *p* = 0.003–0.004); and decreases in the base excess in the blood values (29.24–39.38%, *p* < 0.001) and lactate (13.45–31.97%, *p* < 0.001) in the blood of both breeds in the post-competition horses. Significant increases after competition were determined for the values of creatinine (21.34–30.82%, *p* = 0.001–0.004), total bilirubin (50.84–56.24%, PH < 0.001), and albumin (2.63–4.48%, *p* = 0.048–0.001) for both breeds. For the faster Arabian horse breed, recovering after racing took half the time that the local Žemaitukai breed did.

## 1. Introduction

Fédération Equestre Internationale (FEI) has described equine endurance racing as the second largest discipline in the world, above which is only show jumping [1]. Endurance races are described as long-distance competitions (40–160 km) that are organized into loops, which are arranged over variable terrain, and as a rule, these competitions are completed in one day. In endurance competitions, horse and rider combinations are required to complete the racecourse in good condition, aiming to win [2]. Endurance can be described as an exercise performed at a moderate speed whilst covering a long distance [3]. A horse is an interesting physiological model in this context, because different breeds can be used in all types of physical exercise. For example, the Arabian breed is recognized as well adapted to endurance racing, because the Arabian horses are able to run at an average speed of 20 km/h or greater for up to 160 km (in bouts of 30–40 km) [4]. This level of performance is based on aerobic metabolism, adaptation of the cardiorespiratory system, effective body heat dissipation, and maintenance of homeostasis. The Arabian horse breed is one of the world’s oldest breeds. This breed is distinguished by its natural beauty, graceful movement, and athletic endurance. Since Arabian horses originated from the Middle East, they feature the unique ability to thrive in a hot, dry environment [5]. The standard height of the Arabian horse is from 145 to 155 cm (standing) [6]. The standard weight is between 360 and 450 kg; chest girth—113.2 cm; body length—145 cm; cannon bone girth—18.5 cm. Compared with other race horse breeds, the muscle tissue of Arabian horses is characterized by significant differences in structure—the predominance of oxidative fiber type I is observed in Arabians, [7,8]. In an Arabian horse’s muscle, the higher proportion of oxidative type I fibers (characterized by a low glycogen content and high triglyceride storage capability) results in a greater use of fat for energy [9]. Due to their exceptional qualities, Arabian horses have been extensively used for over 100 years by horse breeders for the improving of other horse breeds [10]. Arabian horses are often used not only for refinement of other horse breeds, but also for their endurance abilities [11]. The studies of the phenotypic traits of the Žemaitukai showed the average body measurements were: wither height—133.6 cm; chest girth—173.2 cm [12]; body length—142.2 cm; cannon bone girth—17.7 cm; weight, 360–420 kg [13]. The Žemaitukai is a pony by height and type [14]. However, the wide chest, high indices of extension, and massiveness indicate that these horses might have substantial draughting power. Due to thin, strong legs, a low bony index, round hips, a wide trot, good jumping technique, energetic temperament, and mobility, the Žemaitukai horses are considered as very suitable for quick trotting and jumping. Thanks to the aforementioned qualities, the Žemaitukai breed is highly valued and used as a versatile horse breed [13]. Based on the results obtained in our last study, it was stated that the horses of the Žemaitukai breed are suitable for endurance competing [15]. To maximize performance during a race, an athlete has to regulate speed over the entire course of a race. The distribution of energy expenditure during the race is defined as “pacing strategy.” Pacing strategy is considered to be a key factor determining overall performance in endurance racing. It is considered that the best strategy in a race is maintaining an even pace, and sometimes gradual declining in speed [16]. The aim of every athlete is to reach a certain distance in the shortest time possible [17]. In endurance athletes, an increased aerobic capacity allows skeletal muscle to metabolize more fat (use energy from fatty acids), while at the same time using carbohydrates as an energy source [18]. An aerobic conditioning program in endurance-type athletes induces an increase in the activity of oxidative metabolism and a decrease in anaerobic metabolism. The adaptive response to exercise is associated with changes in gene expression, metabolism, muscle cell cycle progression, and protein homeostasis. However, the exact mechanisms that occur in equine muscles during exercise related to skeletal muscle endurance in high-intensity training are not well understood [19]. Extensive studies of speed, pace, and total time in racing strategy have been carried out in human sports, whereas in equestrian sport, pacing strategy has been practically ignored, although it is one of the key factors to be analyzed when seeking to optimize horse performance [10]. As regards speed, data on postrace physical, biochemical, and blood gas parameters of endurance horses are limited. Therefore, the aim of this study was to investigate the impacts of breed on speed and the blood parameters of the Arabian and Žemaitukai horses during an endurance race.

## 2. Materials and Methods

### 2.1. Location, Animals, and Experimental Design

The research was carried out in accordance within the provisions of the Law of the Republic of Lithuania—order number 8–500 on the protection, keeping and use of animals, of 6 November 1997 (the Official Gazette “Valstybės žinios” number 108–6595, dated 28 November 1997), order number 4–361 of 31 December 1998 of the State Veterinary Service of the Republic of Lithuania on breeding, care, transportation of laboratory animals, and order number 4 of 18 January 1999 of the State Veterinary Service of the Republic of Lithuania on the use of laboratory animals for scientific tests. The study approval number was PK012868. The study was conducted on 60 (27 female and 33 male) clinically healthy Arabian horses and 52 (34 female and 18 male) clinically healthy Žemaitukai horses at six endurance competitions (45 km races) in Lithuania and at the Lithuanian University of Health Sciences Veterinary Academy. All horses were 10 ± 4 years old, with an average body weight of 408 ± 41 kg. The horses were transported from different locations, having been delivered to the competitions at least two hours prior to their first veterinary examinations. Veterinary inspections were carried out according to FEI regulations. After each loop of the races, all competing horses underwent veterinary inspections, and the physical parameters of each horse were recorded. All horses successfully passed veterinary inspections, which were performed prior to the start of the race, during the race, and after the race. For a horse to be considered fit enough to continue the event, its HR must be below 65 bpm within 20 min of arrival. The criterion for inclusion of horses was successful completion of the race, and only the finishers were included in the further research. All horses were trained for endurance racing and had participated in similar competitions before; however, such factors as the degree of competition experience, the number of competition kilometers completed, and the number of successful races over the recent period were different for each animal. All researched horses were dewormed and vaccinated at a similar time and were not receiving any medications and/or suffering from any infections in the preceding three weeks. All animals were housed in a similar environment and fed a similar diet with adequate mineral and vitamin supplementation; the amounts of salt and water were not limited in the diet.

### 2.2. Analytical Procedures

Blood samples were taken from each animal by applying the technique of jugular venipuncture with 1.6 mL heparinized vacutainer blood collection tubes for blood gas (Terumo Europe, Belgium) and a 5.0 mL tube without anticoagulant for serum biochemistry (Terumo Europe, Belgium), 30 min before the start and no later than 30 min after the finish. None of the horses showed stress during the blood sampling, which was carried out in less than 30 s for each sample. The samples were taken immediately after the first and last veterinary inspections; then they were identified and stored in an ice bath for a maximum period of 30 min until processing. Using EPOC blood gas analyzers (EPOC, Canada, Ottawa), the following indices were analyzed: hydrogen potential (pH), partial carbon dioxide pressure (pCO_2_, kPa), partial oxygen pressure (pO_2_,kPa), base excess in the blood (BE, mmol/L), base excess in the extracellular fluid (BE ecf mmol/L), bicarbonate (HCO_3_, mmol/L), oxygen saturation (sO_2_, %), total carbon dioxide in the blood (tCO_2_, mmol/L), hematocrit (HCT, L/L), hemoglobin concentration (Hgb, mmol/L), glucose (Glu, mmol/L), sodium (Na^+^, mmol/L), potassium (K^+^, mmol/L), calcium (Ca^++^,mmol/L), and lactate (Lac, mmol/L).

Serum urea (Urea, mmol/L), glucose (Glu, mmol/L), aspartate aminotransferase (AST, U/L 1), iron (Fe, umol/L), creatinine (CREA, µmol/L), magnesium (Mg, mmol/L), total protein (TP, g/L), total bilirubin (TB, µmol/L 1), albumin (ALB, g/L), cuprum (Cu, umol/L), zinc (Zn, umol/L), and gamma-glutamyl transferase (GGT, U/L) were measured using an automated analyzer, Hitachi 705 (Hitachi, Japan).

Horse speed and recovery time data were taken from the electronic time-keeping system (ECR v.7.01 Systems, Kaunas, Lithuania) designed for endurance events. Speed data were calculated by dividing the length of the course by the time taken for the horse to complete the course (subtracting hold times). Recovery time is the time that horse spends in the recovery area after crossing the end line of a loop until it crosses the line into the vetting area to be presented for its horse inspection.

The races were held in Lithuania, in accordance with the FEI rules. The competitions were carried out on variable terrain with some muddy and some firm areas, and with slight elevation changes (±300 m). The environmental conditions during the competitions varied, with the mean temperature being 22.5 °C (within the range of 12.50–26.5 °C) and the mean relative humidity being 73.20% (within the range of 52–96%). For a horse to be considered fit enough to continue the event, its HR must be below 65 bpm within 20 min of arrival.

### 2.3. Data Analysis and Statistics

The data were analyzed using the IBM SPSS Statistics v20.5 for Windows. The distributions of the evaluated traits were used to carry out the assessment according to the Kolmogorov–Smirnov test. The mean (M) value and standard error of the mean (SE) were calculated. To compare blood parameters before and after competition, a paired *t*-test was used; to compare the differences between the breeds before and after competition, the Student’s *t*-test for independent samples was employed. The relationships between speed and recovery time and the blood parameters of a horse were assessed using the Spearman method. The results were considered to be significant at *p* < 0.05.

## 3. Results

### 3.1. The Indices of Biochemical Parameters of the Arabian and Žemaitukai Horses before and after Competition

The analysis of biochemical parameters (Table 1) showed significant increases in the values of CREA (21.34–30.82%, *p* = 0.001–0.004), TB (50.84–56.24%, *p* < 0.001), and ALB (2.63–4.48%, *p* = 0.048–0.001) after competition in both breeds.

Before and after competition, significantly higher levels of Fe (*p* = 0.001 and *p* < 0.001), CREA (*p* < 0.001), TP and TB (*p* = 0.023 and *p* < 0.001), and ALB (*p* < 0.001) were observed in the Arabian horses compared to those in the Žemaitukai breed, for which higher AST (*p* < 0.001), Cu (*p* = 0.002 and *p* < 0.001), and GGT (*p* < 0.001) were found.

### 3.2. The Indices of Acid–Base Balance in the Arabian and Žemaitukai Horses before and after Competition

The study showed significant decreases in mean blood gasometrical indicators, such as pCO_2_ (8.09–15.18%, *p* < 0.001) and BE (efc) (14.01%, *p* < 0.001 in the Arabian horses and 172.01% in the Žemaitukai breed, *p* = 0.006) and in the blood electrolyte Ca ++ (4.38–8.72%, *p* < 0.001); and there were increases in HCT and Hgb (20.05–20.12%, *p* < 0.001 in the Arabian horses and 6.22–6.23% in the Žemaitukai breed, *p* = 0.003–0.004), BE (b) (29.24–39.38%, *p* < 0.001), and Lac (13.45–31.97%, *p* < 0.001) in the blood of both breeds in the post-competition horses compared to those measured before competition (Table 2).

### 3.3. Speed and Recovery Time by Breed

This section describes the speed and recovery time after competition for the Arabian and Žemaitukai horses, and the relationships between speed and blood indices. The study showed that based on electronic time-keeping system data, for the faster Arabian horse breed, recovery time was twice as fast, compared to the local Žemaitukai breed (*p* < 0.001) (Table 3).

**Figure 1:**
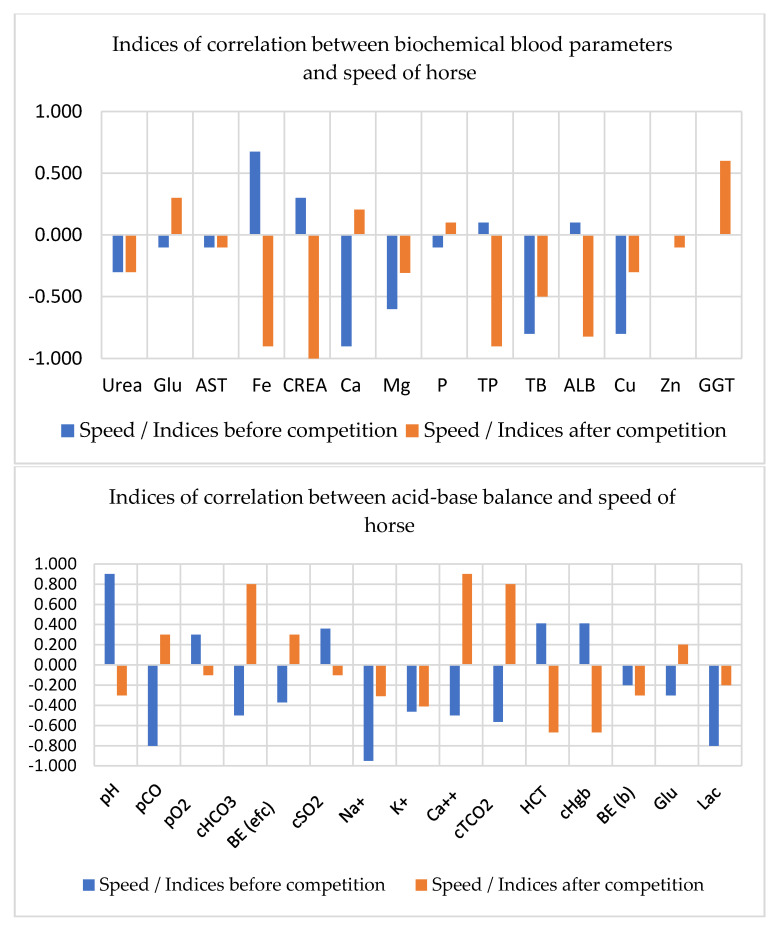
Correlations between blood indices and the speed of the Arabian horses. pH—hydrogen potential; pCO_2_—partial carbon dioxide pressure; pO_2_—partial oxygen pressure; BE—base excess in the blood; BE (efc)—base excess in the extracellular fluid; HCO_3_—bicarbonate; cSO_2_—oxygen saturation; tCO_2_—total carbon dioxide in the blood; HCT—hematocrit; Hgb—hemoglobin concentration; Glu—glucose; Na^+^—sodium; K^+^—potassium; Ca^++^—ionized calcium; Lac—lactate; Urea—serum urea; Glu—glucose; AST—aspartate aminotransferase; Fe—iron; CREA—creatinine; Ca—total calcium; Mg—magnesium; *p*—phosphorus; TP—total protein; TB—total bilirubin; ALB—albumin; Cu—cuprum; Zn—zinc; GGT—gamma-glutamyl transferase.

It was found that of all blood biochemical parameters of the Arabian breed, Fe before competition most positively correlated with the speed of horse (r = 0.675, *p* < 0.01) and GGT (r = 0.600, *p* < 0.01) after competition. Most negatively correlated were Ca, Mg, TB, and Cu (r = 0.600–0.900, *p* < 0.01) before competition; and Fe, CREA, TP, ALB, and GGT (r= 0.600–0.996, *p* < 0.01) after competition (Figure 1). The recovery time of Arabian horses was mainly positively related with blood urea and Zn (r = 0.600–0.800, *p* < 0.01) and negatively correlated with Mg, Cu, and GGT in the blood (r = 0.500–0.975, *p* < 0.01) before and after competition (Figure 1).

Analysis of the correlations between the speed of the Arabian horses (Figure 1E) and the indicators of acid-base balance showed positive correlations with pH, pO_2_, cSO_2_, HCT, and Hgb (*p* < 0.05) and negative correlations with pCO, BE (efc), Na^+^, K^+^, Ca^++^, tCO_2_, Glu, and Lac (*p* < 0.05) before competition. Post-race blood tests for HCO_3_, Ca^++^, and tCO_2_ showed positive correlations of these parameters with the speed of horse, whereas pH, Na^+^, K^+^, HCT, Hgb, and BE (b) negatively correlated with the speed of horse (*p* < 0.05).

**Figure 2:**
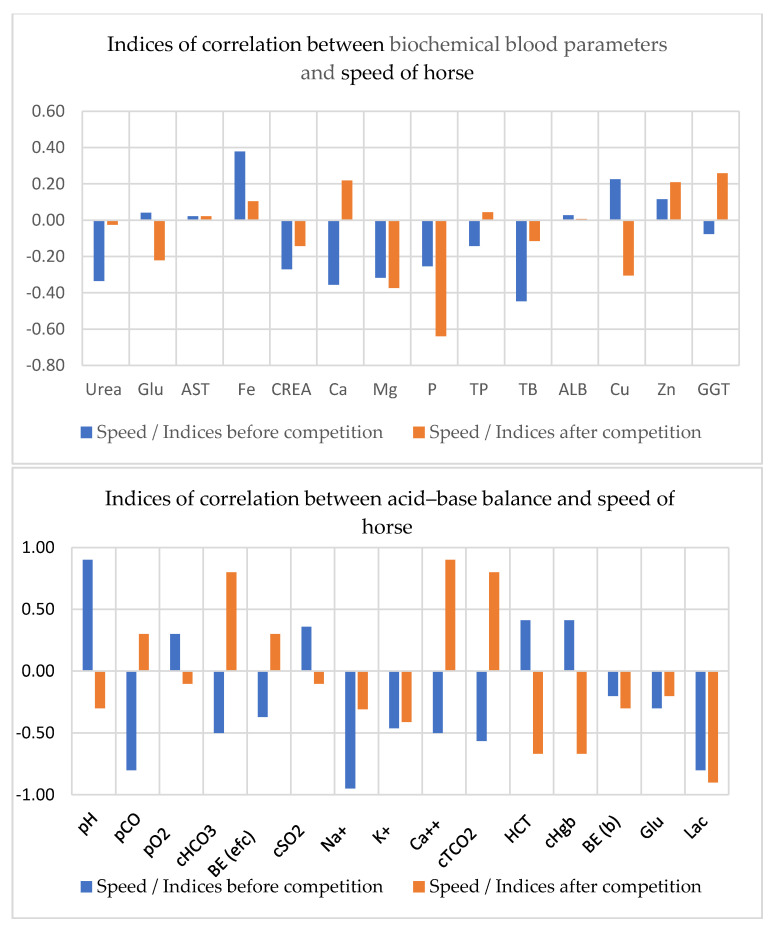
Correlations between blood indices and the speed of the Žemaitukai horses. pH—hydrogen potential; pCO_2_—partial carbon dioxide pressure; pO_2_—partial oxygen pressure; BE—base excess in the blood; BE (efc)—base excess in the extracellular fluid; HCO_3_—bicarbonate; cSO_2_—oxygen saturation; tCO_2_—total carbon dioxide in the blood; HCT—hematocrit; Hgb—hemoglobin concentration; Glu—glucose; Na^+^—sodium; K^+^—potassium; Ca^++^—ionized calcium; Lac—lactate; Urea—serum urea; Glu—glucose; AST—aspartate aminotransferase; Fe—iron; CREA—creatinine; Ca—total calcium; Mg—magnesium; *p*—phosphorus; TP—total protein; TB—total bilirubin; ALB—albumin; Cu—cuprum; Zn—zinc; GGT—gamma-glutamyl transferase.

Based on the correlation analysis of biochemical parameters of the Žemaitukai horses (Figure 2), it can be concluded that *p* before competition and TB after competition were mainly negatively related with the speed of horse (*p <* 0.01). It was also found that the recovery time of this breed (Figure 2) has a negative significant correlation with GGT before competition, and with Mg and *p* after competition (*p <* 0.01).

The indicators of acid–base balance of the Žemaitukai breed showed the highest positive correlation of horse speed with blood pH before competition, and with Ca^++^ (r = 0.900, *p* < 0.01), HCO_3_, and tCO_2_ after competition (r = 0.800, *p* < 0.01). The highest negative correlations of horse speed were with pCO and Lac before competition (r = −0.800–0.802, *p* < 0.01) and with HCT and Hgb after competition (r = −0.670–0.672, *p* < 0.01) (Figure 2).

## 4. Discussion

In the study, significant differences between the pre- and postrace blood parameters of the Žemaitukai and Arabian endurance horses were found. Pre-race blood samples showed lower hematocrit values; however, they still fell within the reference range [23]. In postrace blood testing, an increase of hematocrit was observed. An insignificant increase in hemoglobin was found in postrace blood samples. These findings were described in previous studies [24,25]. Splenic contraction induced by adrenergic stimulus and sweating, causing extensive body fluid losses, has been observed in the conditions of more prolonged exercise [26]. The study revealed relationships between breed and HTC, Hb change, and TP concentration. According to Fan et al. [27], increases in HTC percentage and TP concentration can be indicative of dehydration status occurring in result of the action of xanthine oxidase in free radical production, which in turn is determined by the permeability of muscle cell membrane. Dehydration is normally observed in all horses participating in endurance races. However, dehydration levels may vary. They are directly related to the sweat rate, which is determined by the amount and rate of physical work performed and by the environmental temperature and humidity [28]. Training and heat acclimatization can increase the sweat rate by 10 to 20 percent [29]. Decreased blood volume, high expenditure of energy, and muscular damage can indicate changes in equine biochemical profiles during long endurance racing [30]. Decreased blood volume first of all indicates an extensive loss of body fluid and electrolytes and low intake of fluid. During this study, a negative correlation between the CREA values and horse speed was established for both breeds after competition. Due to muscular metabolism, the final catabolite creatinine is developed, and the final catabolite of endogenous protein breakdown is urea [31]. An elevated concentration of urea can be observed in the horses after prolonged exercise. This was revealed by a study of horses competing in 121 km and 164 km endurance races [32]. This is consistent with the increase in postrace concentration of urea observed in our study. The postrace increase in creatinine concentration was also observed in the horses competing in the 160 km endurance races [33]. Another study revealed that a higher degree of dehydration due to intensified physical effort can result in increased levels of urea and creatinine after the 80 km races [34], implying a significant positive correlation between the above-mentioned parameters. The study showed that CREA levels were increased in both breeds after exercise; however, for the Žemaitukai horses, the rise of creatinine was higher, leading to significant decreases in the urea–CREA ratio. The mechanism for changes in serum creatinine following aerobic training could be related to the theoretical and empirical reports insisting that creatinine concentration is positively associated with body mass index, body fat, and fat distribution [35]. Before and after competition, higher levels of CREA were observed in the Arabian horses, probably as a result of the larger muscle mass [36], although the weights of breeds of horses were similar. A low body fat percentage and a large amount of muscle are of benefit to horses being considered to be elite level endurance racers. Creatinine and urea elevation may also result from a higher metabolic rate [37].

The serum iron concentration was significantly reduced in postrace Žemaitukai horses and increased in the Arabian horses. The ability to maintain prolonged submaximal exercise and the activity of iron-dependent oxidative enzymes is sufficiently closely related to tissue iron levels, which are a determining factor for endurance performance during prolonged submaximal exercise [38]. Lack of hemoglobin may significantly affect physical performance, as there will be a decrease in oxygen transport to exercising muscle. Decreases of hemoglobin levels and tissue iron content may have an adverse effect on performance [38]. Iron supplementation has been reported [39] to increase physical performance and motivation and improve efficient energy use in humans involved in various types of physical activities, whereas iron deficiency contributes to reduced aerobic capacity of muscle, and decreased tissue concentrations of nonheme iron can have detrimental effects because it functions as an enzyme cofactor [40]. Serum concentrations of the iron storage protein ferritin have been correlated with performance in nonanemic female marathon runners [41].

Exercise did not affect mineral requirements greatly, despite the possible increase in the request for minerals associated with the need for more energy to the muscles (Ca, Mg, and *p*) and production of saliva and sweat (Na^+^) [42]. The blood K^+^ levels decreased after racing in both breeds. Similarly, another study found that the loss of potassium via sweat and renal fluid reabsorption through the kidneys were associated with potassium and hydrogen ion release [43]. Di Filippo et al. (2005) [42] also reported that the Arabian horses had a lower K^+^ concentration after 60 km of endurance racing [44]. Lowered K^+^ levels during exercise have a significant negative effect on a horse’s performance [45]. Loss of potassium may result in fatigue, weakness, reduced intestinal motility, and paralytic bowel, and sometimes even lead to altered electrocardiographic traces [46].

In this study, a lactate increase after endurance exercise in the Arabian horses was observed. However, decreased blood lactate concentrations might occur due to poor glucose utilization by the metabolizing tissues [47]. Substitution in the metabolic preference for glucose over lipids was the mainstay of increase in the blood lactate levels. Besides, active exportation of lactate from muscles into the blood is possible [48]. Another study found a sudden upsurge of lactate concentration after 15 min of recovery [49]. The horses with high lactate concentrations showed better results than those with low lactate levels, indicating that other mechanisms may be involved in the regulation of blood lactate concentration [50]. Nevertheless, postrace blood lactate concentrations were higher than pre-race ones for all horses; however, they were elevated more in the Arabian horses than in the Žemaitukai breed, probably owing to the significantly higher anaerobic capacity in the Arabian horses. The postrace values of pH, HCO_3_^−^, BE, and Hct in both horse breeds were higher than the pre-race ones. Increases in pH values of the horses can be related to the loss of chloride ions via sweat. Due to the body’s need to restore the balance of negative charges, the loss of chlorine via sweat results in retention of the second most abundant ions in the organism, bicarbonate ions (HCO_3_^−^) [51]. In turn, the excess of HCO_3_^−^ triggers hypochloremic metabolic alkalosis [45]. According to Johnson (1995), this alkalosis is an important clinical complication in exhaustion syndrome and in the cases of exertional rhabdomyolysis [52]. To maintain pH homeostasis, the body has three lines of response: chemical buffers, respiratory regulation, and renal regulation [43]. Due to changes in the blood pH, respiratory compensation occurs almost immediately, altering the pCO_2_ [52]. Over a long run, regulation of the acid–base balance requires excretion of H^+^ ions and retention of bicarbonate ions by the kidneys [53]. In line with these findings, the results show changes in postrace pCO_2_ and pO_2_. These findings indicated respiratory acidosis, which added to metabolic alteration, is called metabolic alkalosis with respiratory compensation. This respiratory modifications is common in sporting animals when faced with metabolic alkalosis [43], and is a reflection of the animals’ organic health. Increased extraction of oxygen from the blood determines the reduction of venous oxygen content [54]. Under resting conditions, oxygen extraction ranges between 20% and 40%. During exercise, approximately 70–80% of the oxygen delivered to the active muscles may be extracted. This demonstrates that there is a reserve of oxygen in the blood that can be utilized immediately to meet the needs of the contracting muscles at the onset of exercise. Increased extraction of oxygen from the blood is driven by the decreases in perivascular PO_2_, which in turn are driven by the reductions in cell PO_2_ [9]. The apparent breed difference in aerobic and anaerobic capacity may, in part, reflect breed variation in muscle fiber types and the muscle concentrations and activities of enzymes involved in glycolysis [55]. Excess body fat increases the energy requirements of weightbearing work, such as running, by increasing the energy requirements of exercise for any given intensity of work during a maximal oxygen consumption test [56]. This may be detrimental to running performance in that the running speed that can be sustained for a given duration is reduced [57], thereby increasing race time.

## 5. Conclusions

This study showed that for the faster Arabian horse breed, recovering after racing took half the time taken by the local Žemaitukai breed. Based on hematocrit changes during exercise, the Arabian horses were found to have a higher sweat rate. Before and after competition, higher levels of creatinine were observed in the Arabian horses, probably as a result of having more muscle mass than Zemaitukai horses. Postrace blood lactate concentrations were higher for all horses; however, they were elevated more in the Arabian horses because of the greater muscle mass and higher activity level of the latter breed. Lower amounts of electrolytes and smaller changes during races showed that the Žemaitukai horses had a lower capacity for heat tolerance, suggesting that the Žemaitukai horses were less trained for endurance competition. Those differences between the two breeds might be based on different amounts of fat free body mass. Further studies are required to determine the inter-breed differences in muscle architecture and body composition.

## Figures and Tables

**Table 1:** The indices of minerals, trace elements, and biochemical parameters in the blood of the Arabian and Žemaitukai horses before and after competition.

Blood Parameter	Breed	Pre-Race			Post-Race			Changes (Pre-Race—Postrace)		Normal Ranges [20]
		M	SE	*p*	M	SE	*p*	Difference (%)	*p*	
Urea mmol/L	Arabian	6.06	0.181	<0.001	7.29	0.311	0.675	20.30	<0.001	2.9–9.6
	Žemaitukai	7.05	0.181		7.11	0.311		0.85	0.802	
AST U/L	Arabian	301.02	12.403	<0.001	336.93	11.862	<0.001	11.93	<0.001	205–555
	Žemaitukai	434.36	12.403		410.09	11.862		−5.59	0.027	
Fe umol/L	Arabian	31.34	0.849	<0.001	32.99	0.920	<0.001	5.25	0.127	20–45
	Žemaitukai	27.52	0.849		25.60	0.920		−7.00	0.002	
CREA µmol/L	Arabian	133.00	2.539	<0.001	161.38	3.012	<0.001	21.34	0.001	53.1–159.2
	Žemaitukai	107.77	2.539		144.15	3.012		30.82	0.004	
Mg mmol/L	Arabian	0.75	0.031	0.336	1.42	0.248	0.048	89.33	0.058	0.6–1.7
	Žemaitukai	0.71	0.031		0.72	0.248		1.41	0.892	
anorg P mmol/L	Arabian	1.03	0.250	0.994	1.05	0.249	0.958	−1.28	0.006	0.8–1.3
	Žemaitukai	1.04	0.250		1.03	0.249		0.85	0.492	
Total Ca mmol/L	Arabian	0.67	0.036	<0.001	0.67	0.030	<0.001	0.00	0.953	0.59–0.74
	Zemaitukai	0.76	0.036		0.76	0.030		0.00	0.874	
TP g/L	Arabian	78.77	0.870	0.023	83.62	1.098	<0.001	6.16	<0.001	56–76
	Žemaitukai	75.93	0.870		75.63	1.098		−0.40	0.643	
TB (bilirub) µmol/L	Arabian	31.61	1.467	<0.001	47.68	2.095	<0.001	50.84	<0.001	25–42
	Žemaitukai	15.95	1.467		24.92	2.095		56.24	<0.001	
ALB g/L	Arabian	41.28	0.336	<0.001	43.13	0.417	<0.001	4.48	0.001	26–41
	Žemaitukai	36.93	0.336		37.90	0.417		2.63	0.048	
Cu umol/L	Arabian	10.40	1.141	0.002	10.91	1.428	0.001	4.92	0.177	7.9–39.5
	Žemaitukai	12.61	1.141		13.41	1.428		6.39	0.122	
Zn umol/L	Arabian	26.99	0.817	0.010	28.07	0.683	0.230	3.98	0.112	12–45
	Žemaitukai	28.64	0.817		27.62	0.683		−3.56	0.001	
GGT U/L	Arabian	17.18	2.328	<0.001	16.70	2.390	<0.001	−2.79	0.353	6–32
	Žemaitukai	34.13	2.328		32.70	2.390		−4.19	<0.001	

M—mean; *p*—calculated probability; SE—standard error of the mean; Urea—serum urea; AST—aspartate aminotransferase; Fe—iron; CREA—creatinine; Ca—calcium; Mg—magnesium; TP—total protein; TB—total bilirubin; ALB—albumin; Cu- cuprum; Zn—zinc; GGT—gamma-glutamyl transferase; ND—no data.

**Table 2:** The indices of electrolyte and acid–base balance in the Arabian and Žemaitukai horses before and after competition.

Blood Parameter	Breed	Pre-Race			Post-Race			Changes (Pre-Race—Postrace)		Normal Ranges of Venous Blood [21,22]
		M	SE	*p*	M	SE	*p*	Difference (%)	*p*	
pH	Arabian	7.45	0.005	<0.001	7.51	0.005	<0.001	0.81	<0.001	7.36–7.43
	Žemaitukai	7.49	0.007		7.53	0.007		0.53	<0.001	
pCO_2_ kPa	Arabian	5.34	0.094	<0.001	4.53	0.076	<0.001	−15.18	<0.001	5.06–6.39
	Žemaitukai	4.75	0.116		4.36	0.094		−8.09	<0.001	
pO_2_ kPa	Arabian	4.91	0.342	0.016	4.65	0.121	0.016	−5.29	0.721	4.8–6.13
	Žemaitukai	6.59	0.660		5.39	0.386		−18.13	0.037	
HCO_3_ mmol/L	Arabian	27.66	0.311	0.032	27.92	0.413	0.032	0.94	0.615	22–29
	Žemaitukai	26.58	0.384		26.89	0.510		1.17	0.452	
BE (efc) mmol/L	Arabian	3.64	0.295	0.333	4.15	1.090	0.333	14.01	<0.001	0–5
	Žemaitukai	3.18	0.364		8.65	1.348		172.01	0.006	
sO_2_ %	Arabian	75.24	0.544	0.001	75.99	0.618	0.001	0.99	0.320	70–75
	Žemaitukai	78.23	0.673		77.24	0.764		−1.26	0.085	
Na^+^ mmol/L	Arabian	139.69	0.254	0.539	139.23	0.209	0.539	−0.33	0.057	136–142
	Žemaitukai	139.94	0.314		137.76	0.259		−1.56	<0.001	
K^+^ mmol/L	Arabian	3.54	0.048	0.158	3.28	0.053	0.158	−7.34	<0.001	2.20–4.60
	Žemaitukai	3.65	0.059		3.58	0.065		−1.92	0.577	
Ca^++^ mmol/L	Arabian	1.49	0.010	<0.001	1.36	0.011	<0.001	−8.72	<0.001	1.25–1.75
	Žemaitukai	1.37	0.013		1.31	0.014		−4.38	<0.001	
tCO_2_ mmol/L	Arabian	28.88	0.329	0.026	28.94	0.379	0.026	0.21	0.892	22–33
	Žemaitukai	27.69	0.406		27.93	0.469		0.87	0.376	
HCT L/L	Arabian	0.37	0.069	0.062	0.44	0.059	0.062	20.12	<0.001	30–45
	Žemaitukai	0.35	0.085		0.37	0.074		6.23	0.003	
Hgb mmol/L	Arabian	7.83	0.140	0.054	9.4	0.130	0.054	20.05	<0.001	6.21–9.31
	Žemaitukai	7.38	0.187		7.84	0.155		6.22	0.004	
BE mmol/L	Arabian	3.42	0.248	0.586	4.42	0.157	0.586	29.24	<0.001	0–5
	Žemaitukai	3.20	0.307		4.46	0.194		39.38	<0.001	
Glu mmol/L	Arabian	5.80	0.116	0.004	6.09	0.159	0.004	5.00	0.056	3.4–7.4
	Žemaitukai	5.26	0.143		5.85	0.196		11.22	0.001	
Lac mmol/L	Arabian	1.47	0.098	0.121	1.94	0.116	0.121	31.97	<0.001	<2.5
	Žemaitukai	1.71	0.121		1.94	0.143		13.45	<0.001	

M—mean; *p*—calculated probability; SE—standard error of the mean; pH—hydrogen potential; pCO_2_—partial carbon dioxide pressure; pO_2_—partial oxygen pressure; BE—base excess in the blood; BE (efc)—base excess in the extracellular fluid; HCO_3_—bicarbonate; sO_2_—oxygen saturation; tCO_2_—total carbon dioxide in the blood; HCT—hematocrit; Hgb—hemoglobin concentration; Glu—glucose; Na^+^—sodium; K^+^—potassium; Ca^++^—ionized calcium; Lac—lactate; ND—no data.

**Table 3:** Speed and recovery time by breed after 45 km ride.

Indicator	Breed	*n*	M	SE
Speed km/h	Arabian	52	15.030	0.195
	Žemaitukai	60	14.039 ***	0.181
Total time (hh:mm:ss)	Arabian	52	2:57:47	0:05:32
	Žemaitukai	60	3:12:02	0:05:24
Recovery time (hh:mm:ss)	Arabian	52	0:04:41	0:00:33
	Žemaitukai	60	0:09:10 ***	0:00:57

*** *p* < 0.001; *n*—total number of horses; M—mean; SE—standard error of the mean, km/h—kilometers per hour.

## Data Availability

The data presented in this study are available within the article.
